# Use of Go-Beyond as a Self-Directed Internet-Based Program Supporting Veterans’ Transition to Civilian Life: Preliminary Usability Study

**DOI:** 10.2196/60868

**Published:** 2025-01-23

**Authors:** Karolina Katarzyna Alichniewicz, Sarah Hampton, Madeline Romaniuk, Darcy Bennett, Camila Guindalini

**Affiliations:** 1 Greenslopes Private Hospital Gallipoli Medical Research Brisbane Australia; 2 The University of Queensland Faculty of Health and Behavioural Sciences Brisbane Australia; 3 School of Medicine The University of Queensland Brisbane Australia

**Keywords:** military transition, web-based interventions, military-civilian adjustment, Go-Beyond, internet-based program, civilian, military service, veteran, premilitary life, mental health issues, physical injuries, adoption, quantitative analysis, survey, family, support, digital technology, user engagement, effectiveness, assessment

## Abstract

**Background:**

The transition from military service to civilian life presents a variety of challenges for veterans, influenced by individual factors such as premilitary life, length of service, and deployment history. Mental health issues, physical injuries, difficulties in relationships, and identity loss compound the reintegration process. To address these challenges, various face-to-face and internet-based programs are available yet underused. This paper presents the preliminary evaluation of “Go-Beyond, Navigating Life Beyond Service,” an internet-based psychoeducational program for veterans.

**Objective:**

The study aims to identify the reach, adoption, and engagement with the program and to generate future recommendations to enhance its overall impact.

**Methods:**

This study exclusively used data that were automatically and routinely collected from the start of the Go-Beyond program’s launch on May 24, 2021, until May 7, 2023. When accessing the Go-Beyond website, veterans were asked to complete the Military-Civilian Adjustment and Reintegration Measure (M-CARM) questionnaire, which produces a unique M-CARM profile of results specifying potential areas of need on the 5 domains of the measure. Users were then automatically allocated to Go-Beyond modules that aligned with their M-CARM profile. Additionally, quantitative and qualitative data were collected from a survey on aesthetics, interactivity, user journey, and user experience, which was optional for users to complete at the end of each module.

**Results:**

Results show a conversion rate of 28.5% (273/959) from the M-CARM survey to the Go-Beyond program. This rate is notably higher compared with similar internet-based self-help programs, such as VetChange (1033/22,087, 4.7%) and resources for gambling behavior (5652/8083, 14%), but lower than the MoodGYM program (82,159/194,840, 42.2%). However, these comparisons should be interpreted with caution due to the limited availability of published conversion rates and varying definitions of uptake and adoption across studies. Additionally, individuals were 1.64 (95% CI 1.17-2.28) more likely to enroll when they express a need in Purpose and Connection, and they were 1.50 (95% CI 1.06-2.18) times more likely to enroll when they express the need Beliefs About Civilians, compared with those without these needs. The overall completion rate for the program was 31% (85/273) and modules’ individual completion rates varied from 8.4% (17/203) to 20% (41/206). Feedback survey revealed high overall user satisfaction with Go-Beyond, emphasizing its engaging content and user-friendly modules. Notably, 94% (88/94) of survey respondents indicated they would recommend the program to other veterans, family, or friends.

**Conclusions:**

The Go-Beyond program may offer promising support for veterans transitioning to civilian life through digital technology. Our study reveals insights on user engagement and adoption, emphasizing the need for ongoing evaluation to further address the diverse needs of military personnel. Future research should explore predictors of engagement, the addition of peer or facilitator support, and the use of outcome measures for effectiveness assessment.

## Introduction

The transition from military service to civilian life is a multifaceted process that can present challenges for veterans [1-3]. Upon leaving the military, veterans often face a period of adjustment as they navigate a vastly different sociocultural and professional landscape [4-6]. The process of transitioning from military service to civilian life is not uniform, as each veteran’s experience is shaped by individual factors such as premilitary life, length of service, deployment history, and personal resilience [7]. While many individuals successfully navigate this change, a substantial proportion experience difficulty in adjusting to civilian life, with these difficulties persisting over the years [1,2,4].

In the literature, there is recognition of various challenges faced by military personnel when they transition into civilian life. Among these challenges, mental health issues such as posttraumatic stress disorder (PTSD), substance use disorders, depression, and anxiety are frequently reported [1]. In addition, physical injuries, including chronic pain and traumatic brain injuries, play a significant role [8]. Furthermore, difficulties in establishing and maintaining friendships and family relationships [4,5], loss of identity and purpose [2,3,9], disparities between military and civilian cultures, as well as employment factors [6] are all critical elements. Collectively, these multiple and dynamic factors have a significant impact on the reintegration of veterans into civilian life. It is important to note that the presence of hardships in various domains can compound the effects and exacerbate functional impairments beyond those caused by individual stressors alone [10]. Consequently, understanding the factors that influence transition and identifying effective accessible interventions is critical in supporting military personnel during this crucial phase.

Internet-based approaches can complement and expand the support systems available to service members and veterans [11-13]. By harnessing the potential of digital technology, personalized and accessible interventions that cater to the diverse needs of military personnel during their transition to civilian life can be provided. Moreover, internet-based interventions could present an effective way to overcome some of the barriers to care, such as long waiting periods, stigma, or lack of access to treatment due to remote location [12]. Internet-based interventions hold significant potential in supporting veterans during the critical transitioning phase. However, ensuring their uptake and effectiveness requires robust evaluations, covering aspects like utility, user satisfaction, usability, acceptability, and potential in addressing the diverse needs of transitioning military personnel [14]. Through such evaluations, policy makers, researchers, and support organizations can gain valuable insights into the most beneficial strategies for veterans [15]. Furthermore, a comprehensive assessment of internet-based interventions may enable tailored interventions, improvement of support mechanisms, and the development of evidence-based practices [16]. Therefore, understanding the impact of internet-based interventions is paramount in enhancing the well-being and successful integration of military veterans into civilian society.

In this paper, we present the preliminary evaluation of an internet-based psychoeducational program “Go-Beyond, Navigating Life Beyond Service” designed for veterans who are in the process of reintegration to civilian life. The aim of the study was to perform a user-centered evaluation of the Go-Beyond program using user engagement data routinely collected and assess the reach, adoption, engagement, and completion rates. Furthermore, the study aimed to gather user feedback to enhance the concept version of the program’s modules, identify opportunities for further improvement, and generate future recommendations.

## Methods

### Program Description

“Go-Beyond, Navigating Life Beyond Service” is an evidence-informed, self-directed, web-based program designed to support veterans’ adjustment to civilian life after separation from military service. The program was developed by Gallipoli Medical Research (GMR), a not-for-profit organization that has the mission to improve the quality of life for veterans and their families through research, translation, and implementation, in partnership with Returned and Services League of Australia Queensland (RSLQ), and was officially launched on May 24, 2021. Over the 24 months after the launch, all interested veterans were provided unlimited access to the Go-Beyond program. Former service members were made aware of the Go-Beyond program through a national implementation task force initiative that included flyers, posters, presentations, and a social media campaign and was conducted by GMR, RSLQ, Department of Veteran Affairs, Open Arms, and Commonwealth Super Corporation. The program was also promoted to various ex-serving organizations. Furthermore, Go-Beyond was accompanied by an applied practitioner portal that provided training on the use of the program and targeted clinicians, especially rehab providers. The implementation of the Go-Beyond program was part of a larger research agreement with RSLQ, and the associated costs for its development and preliminary implementation have been covered by the funding organization. This funding covered essential elements such as platform design, content creation, technical support, and user testing to ensure the program’s accessibility for veterans. In addition, future strategies are being developed to ensure the program’s sustainability beyond the funding period in Australia.

When accessing the Go-Beyond website, veterans are asked to complete the Military-Civilian Adjustment and Reintegration Measure (M-CARM) questionnaire, which produces a unique M-CARM profile of results specifying potential areas of need on the 5 factors of the measure. The M-CARM is a valid and reliable self-report questionnaire designed to assess adjustment and reintegration to civilian life after service. It contains 21 questions, rated from 1 to 5, and takes approximately 5 minutes to complete. The measure is freely available on a dedicated website [17]. Users are automatically allocated to Go-Beyond modules that align with their M-CARM profile.

The Go-Beyond program aims to (1) provide education about topics that are important for psychological adjustment and reintegration to civilian life; (2) build psychological insight into personal challenges adjusting to civilian life; (3) offer practical first steps and tools to facilitate stronger adjustment and reintegration; and (4) provide links to additional services and support available in Australia.

The Go-Beyond program was developed by 2 clinical psychologists at GMR who have both research and clinical experience with ex-service personnel in Australia. The content of the program is based on previous research describing empirically derived psychological and cultural factors associated with adjustment and reintegration to civilian life, including (1) Purpose and Connection, (2) Help Seeking, (3) Beliefs About Civilians, (4) Resentment and Regret, and (5) Regimentation [18]. These factors were derived as part of the development of the M-CARM [18]. Go-Beyond consists of seven interactive modules entitled (1) Finding Purpose, (2) Help Seeking, (3) Regimentation, (4) Resentment and Regret, (5) Social Connection, (6) Beliefs About Civilians, and (7) an optional module available to anyone who signed up to Go-Beyond: (7) Effective Communication in Civilian Life. They are presented to participants in the above order. If a participant is not required to do a particular module, it gets omitted in that order. The session outline of the program is presented in Textbox 1. The program content uses evidence-based psychological therapeutic approaches including cognitive behavioral therapy and acceptance and commitment therapy. Both cognitive behavioral therapy and acceptance and commitment therapy have demonstrated transdiagnostic treatment efficacy within veteran populations [19]. Each module includes (1) psychoeducation for each topic; (2) hypothetical case studies and personal, video-recorded stories from Australian ex-service members; (3) personal reflection opportunities or written tasks; (4) helpful activities; and (5) direction to more specific services and resources for Australian ex-service personnel. The approximate time needed to complete each module is 45-60 minutes.

**Descriptions of Go-Beyond modules.**

**Beliefs About Civilians**

- The module introduces the concept of beliefs, social identity theory, and common unhelpful beliefs about civilians that can hinder adjustment to civilian life. Participants engage in self-reflection to evaluate their beliefs and learn techniques to adapt them, including thought challenging, defusing exercises, and a “take your thought to court” activity. The importance of practicing these techniques is emphasized throughout.

**Finding Purpose**

- The module begins by exploring the importance of purpose, particularly in the military context, and the sense of loss that can occur during the transition to civilian life. It helps participants clarify their values, distinguish between values and goals, and engage in activities like the life compass, values-guided actions, and goal setting. The module also emphasizes finding purpose through volunteering or paid employment and provides information on services and resources that support veterans in securing meaningful work.

**Help Seeking**

- The module provides psychoeducation on the importance of help-seeking for mental health, addressing common barriers and highlighting the rates of help-seeking among Australian veterans. It covers how to find evidence-based treatments and recognize when help is needed. Through the “Staying Well” activity, participants learn to identify indicators of good mental health, and signs of decline, and create an action plan. Practical steps for accessing high-quality mental health support are also provided.

**Social Connections**

- This module covers the importance of social connections for health and well-being, focusing on their role in adjusting to transitions, especially for the “Defence family.” It addresses common barriers such as cultural differences, misconceptions, and communication issues. Strategies for finding mateship outside the military, strengthening existing ties, and building new ones are discussed, alongside opportunities for social connection and volunteering.

**Resentment and Regret**

- This module explores resentment, covering its definition, functions, and impacts, and includes activities to identify underlying feelings, use self-compassion, and resolve related injustices. It also addresses regret, discussing its nature, impacts, and irrationality, with activities to manage regret by confronting hindsight bias, accepting imperfections, and clarifying values and goals.

**Regimentation**

- This module covers regimentation; its role in Defence; and its effects on personal life, family, and work. It includes activities to identify and evaluate regimentation’s impact and emphasizes the importance of psychological flexibility and adaptability. Practical strategies are provided for embracing change, trying new things, and adjusting routines and thought processes.

**Effective Communication in Civilian Life**

- This module covers communication styles (aggressive, passive, passive-aggressive, and assertive) and includes a quiz to identify your style. It provides skills for meaningful conversations, including asking questions, self-disclosure, managing stereotypes, active listening, and overcoming barriers. The module also explores using compliments and empathy to build social connections.

### Data Collection

This study exclusively used data that were automatically and routinely collected starting from the program’s launch on May 24, 2021, until May 7, 2023, through websites [17] for M-CARM and [20] for Go-Beyond. Both websites were freely available to everyone, both veterans and civilian populations, in Australia. No specific inclusion or exclusion criteria were applied to this study. The reach of Go-Beyond was defined as the number of users who accessed and completed the M-CARM measure and the adoption was defined as the number of users who signed up to the Go-Beyond program. Completion was measured by the number of users who accessed and reached the end of the module, as tracked by the Absorb LMS system (Absorb Software Inc). The system records whether a user has started, is in progress, and has fully completed the module, including exiting the module properly. The modules were designed to be completed in 45-60 minutes.

In addition, quantitative and qualitative data were collected from a survey on aesthetics, interactivity, user journey, and user experience, which was optional for users to complete at the end of each module. The questions asked can be found in Table 1.

**Table 1:** User experience survey.

Question	Rating options
How did you find the duration of the module?	Option for comments
How easy was the Go-Beyond Platform to use? Were you able to navigate to what you needed? Please provide details.	Option for comments
How likely are you to implement changes in your life following this module?	Likert scale 1-5 1-Very Unlikely 2-Unlikely 3-Likely 4-Very Likely 5-Extremely Likely
If yes (likely as above), what changes would you make? If no (unlikely or very unlikely), why not?	Option for comments
How many minutes did it take for you to complete this module?	Option for comments
How useful did you find this module?	Likert scale 1-5 1-Completely dissatisfied 2-Dissatisfied 3-Neutral 4-Satisfied 5-Completely satisfied
How would you rate the aesthetics (layout, graphics, or visuals)?	Likert scale 1-5 1-Completely dissatisfied 2-Dissatisfied 3-Neutral 4-Satisfied 5-Completely satisfied
How would you rate the gestural design (interactions, taps, swipes, and scrolling) functions?	Likert scale 1-5 1-Completely dissatisfied 2-Dissatisfied 3-Neutral 4-Satisfied 5-Completely satisfied
How would you rate the performance of the module (features and components)?	Likert scale 1-5 1-Completely dissatisfied 2-Dissatisfied 3-Neutral 4-Satisfied 5-Completely satisfied
How would you rate the quality of content?	Likert scale 1-5 1-Completely dissatisfied 2-Dissatisfied 3-Neutral 4-Satisfied 5-Completely satisfied
How would you rate the usability or navigation of this module?	Likert scale 1-5 1-Completely dissatisfied 2-Dissatisfied 3-Neutral 4-Satisfied 5-Completely satisfied
Overall how engaging did you find the module (entertainment, interest, relatability, and interactivity)?	Likert scale 1-5 1-Completely dissatisfied 2-Dissatisfied 3-Neutral 4-Satisfied 5-Completely satisfied
Overall what would you rate this module?	Likert scale 1-5 1- Poor 2-Satisfactory 3-Good 4-Very Good 5-Excellent
What device did you use to access the module (desktop, mobile, or tablet)?	Options to choose from: Laptop Mobile Tablet Desktop Combination
Would you recommend this module to other veterans or family or friends of a veteran?	Options to choose from: Yes, No
Did the audio and visual presentations resonate with you and your personal experiences?	Options to choose from: No, Somewhat, Yes, No comment
How did you find the use of personal stories, videos, animations, and illustrations?	Option for comments
Please make any further comments regarding the engagement and communication of the module.	Option for comments
Please provide any additional comments on the functionality of the module.	Option for comments
Was the visual information accurate (concepts are logical and correct)? Please provide any comments.	Option for comments
What further recommendations or comments would you make to improve the Go-Beyond Platform and the module?	Option for comments
What was the least helpful part of the module?	Option for comments
What was the most helpful part of the module?	Option for comments

### Statistical Analysis

To determine the reach and adoption of the Go-Beyond program, a user-centered evaluation was performed using information routinely collected to monitor and improve the quality of the program. The system engagement data were automatically collected from users who accessed the M-CARM and Go-Beyond websites on a voluntary basis.

The data analysis was conducted using R (R Core Team) [21]. Descriptive statistics were used to gain insights into the distribution and central tendencies of the collected data. Subsequently, logistic regression analysis was used to discern factors influencing users’ likelihood to progress to Go-Beyond following completion of the M-CARM.

To analyze the qualitative data gathered through the voluntary online surveys, a systematic approach was used. Thematic analysis was used to recognize patterns and identify core themes. Themes were extracted from the data and grouped under the questions posed adhering to the thematic analysis approach by Braun and Clarke [22]. The initial coding of responses was conducted manually by the primary researcher, who independently reviewed the data to develop preliminary codes. Responses were coded into categories and were organized thematically, allowing for the identification of recurring topics. These codes were then reviewed and refined through consensus discussions among the research team, ensuring that differing perspectives were considered. The researchers acknowledge that their perspectives and experiences may influence their interpretations, and they have taken steps to reflect on and mitigate potential biases throughout the research process. The positionality of the researchers was acknowledged throughout the analysis process, with the team reflecting on how their backgrounds, experiences, and potential biases might influence the interpretation of the data and this qualitative analysis [23]. All researchers were committed to evaluating a web-based intervention aiming for it to be not only useful but also feasible and acceptable to the intended users. The primary researcher brings over 10 years of experience working with clients from military backgrounds, providing a unique perspective informed by extensive professional practice. However, it is important to note that the primary and senior researchers were not involved in the creation or implementation of the version of the program under study, positioning them as outsiders in this context. This outsider status allows for a more detached analysis, free from potential biases related to program design and execution. Consensus discussions among the research team also ensured that individual biases were minimized and enhanced the credibility of the interpretations. Representative quotations and exemplars were extracted to illustrate each identified theme.

### Ethical Considerations

The Department of Defense and Veterans’ Affairs Human Research Ethics Committee has deemed this study to be a quality assurance activity that upholds the principles of the National Statement on Ethical Conduct in Human Research and the Ethical Consideration in Quality Assurance and Evaluation Activities (approval number 516-23). In the privacy policy statement that participants have access to when deciding to sign in to the program, they are informed about the organization’s commitment to comply with the Privacy Act 1988 (Cth) (Privacy Act), including the Australian Privacy Principles. The data were extracted from the Absorb LMS system by the LMS Administrator and deidentified before they were provided to the research team. After data cleaning and integration, analysis was conducted by the research team on deidentified data. All collected data were held confidential at all times, in a password-protected database, on a server that requires a unique log-in and password by all team members. Only the project team had access to the data. No compensation was provided to users as this service evaluation used routinely collected data.

## Results

### Go-Beyond User and M-CARM–Only User Profile

Out of the total of 959 users who voluntarily completed M-CARM, there were 686 who did not proceed to sign up for the Go-Beyond program (M-CARM–only users) and 273 who signed up for Go-Beyond, resulting in a conversion rate of 28.5% (273/959) and a preprogram dropout rate of 71.5% (686/959). Table 2 summarizes the demographic characteristics of both user groups. Only upon signing up for the Go-Beyond program, the data regarding time since discharge from the Australian Defence Force were recorded. Out of 273 Go-Beyond users, 215 were discharged from the Australian Defence Force and 43 reported that they were still serving. The median number of years since transition for Go-Beyond users who were discharged equaled 2.74 years, with a large proportion of users (165/273, 60.5 %) reporting being within 0-5 years from discharge.

**Table 2:** Demographic characteristics of the Go-Beyond users and Military-Civilian Adjustment and Reintegration Measure (M-CARM)–only users.

Variables		Go-Beyond users (n=273)	M-CARM–only users (n=686)
**Sex, n (%)**			
	Male	192 (70.3)	498 (72.6)
	Female	67 (25)	178 (26)
	Prefer not to answer	1 (0.2)	10 (2)
	N/A^a^	13 (4.8)	(0)
**Age range (years), n (%)**			
	18-25	20 (7.3)	74 (11)
	26-35	65 (24)	187 (27.3)
	36-45	59 (21)	162 (23.6)
	46-55	73 (26.7)	144 (21)
	56-65	32 (12)	93 (14)
	66-75	8 (3)	15 (2)
	>75	2 (1)	6 (1)
	Prefer not to answer	1 (0.2)	5 (1)
	N/A	13 (4.8)	(0)
**Service, n (%)**			
	Army	153 (56)	384 (56)
	Navy	48 (18)	96 (14)
	Airforce	31 (11)	117 (17)
	Prefer not to answer	24 (9)	77 (11)
	Army+Airforce	2 (1)	3 (0.4)
	Army+Navy	1 (0.2)	1 (0.2)
	Navy+Airforce	0 (0)	2 (0.3)
	N/A	14 (5.1)	0 (0)
**Type of needs as shown on M-CARM, n (%)^b^**			
	Beliefs About Civilians	197 (75.5)	441 (64.3)
	Regimentation	182 (69.7)	452 (65.9)
	Resentment and Regret	176 (67.4)	396 (57.7)
	Purpose and Connection	146 (55.9)	287 (41.8)
	Help Seeking	76 (29.1)	184 (26.8)

^a^N/A: not applicable.

^b^One person can have multiple needs.

There were no statistically significant differences observed between the Go-Beyond and M-CARM–only user groups concerning age, sex, or service type (all *P*>.05). However, a significantly higher number of individuals in the Go-Beyond user group were identified as having needs in the areas of Purpose and Connection (*χ*²_4_=14.59, *P*<.001), Resentment and Regret (*χ*²_4_=7.05, *P*=.008), and Beliefs About Civilians (*χ*²_4_=10.273, *P*=.001) compared with the M-CARM–only users.

A logistic regression was then performed to assess the effects of M-CARM outcomes and service type on the likelihood of enrolling in the Go-Beyond program. Participant’s age, sex, and service type were controlled for. The outcomes of logistic regression showed that participants with the need for Purpose and Connection and Beliefs About Civilians were found to be significantly more likely to sign up for Go-Beyond (*P*=.003 and *P*=.03 respectively). Individuals expressing a need for Purpose and Connection were approximately 1.64 times more likely to enroll (odds ratio 1.64, 95% CI 1.17-2.28) compared with those who did not. Similarly, those with the need Beliefs About Civilians were 1.50 times more likely to enroll (odds ratio 1.50, 95% CI 1.06-2.18) compared with those without this need. Individuals who were identified with a need for Resentment and Regret were not found to be significantly more likely to sign up for Go-Beyond (*P*>.05).

### Go-Beyond User Engagement

Out of 273 users who signed up for Go-Beyond, 85 (31%) users completed at least 1 assigned module. The data on the enrollment status at the time of the data extraction for each of the modules and corresponding completion rates can be found in Table 3. Out of participants who completed at least 1 module, nearly all users who had been assigned 1 module, completed it (36/38, 94% users). Among users who were assigned 2 modules, 37% (14/38) have completed them both. For those who had been assigned 3 modules, 26% (9/34) have completed all 3. Of users who had been assigned 4 modules, 10% (3/31) have completed them, and of users who had been assigned 5 modules, 3% (2/63) have completed all 5. A total of 22% (10/45) of users who had assigned 6 modules have completed all 6. Overall, 3% (8/247) of participants who had been assigned other modules also completed the Communication module. Out of Go-Beyond users, who did not have any modules assigned, but still accessed the program (26 users), 4 (15%) users completed 1 module, 1 (4%) user completed 3 modules, 2 (8%) users completed 4 modules, 1 (4%) user completed all available modules, and 3 (12%) completed the Communication module. The module with the highest completion rate was Finding Purpose with 41 (20%) out of 206 users who signed up to this module, followed by Beliefs About Civilians with 48 (19.7%) out of 244 who signed up, Resentment and Regret with 38 (17.6%) out of 216 who signed up, Regimentation with 33 (16%) out of 205 who signed up, Help Seeking with 21 (15.5%) out of 156 who signed up, and Social Connection with 17 (8.4%) out of 220 signed up users. The lowest completion rate was the additional module Effective Communication in Civilian Life, in which everyone was enrolled automatically, with 11 (4%) out of 273 users who signed up.

**Table 3:** Enrollment status for each Go-Beyond module at the time of data extraction.

Module and status	Not started, n (%)	In progress, n (%)	Complete, n (%)
Beliefs About Civilians (n=244)	151 (61.9)	45 (18.4)	48 (19.7)
Finding Purpose (n=206)	128 (62)	37 (18)	41 (20)
Resentment and Regret (n=216)	163 (75.5)	15 (6.9)	38 (17.6)
Regimentation (n=205)	166 (81)	6 (3)	33 (16)
Help Seeking (n=156)	110 (81.5)	4 (3)	21 (15.5)
Social Connection (n=220)	175 (86)	11 (5.4)	17 (8.4)
Communication^a^ (n=273)	262 (96)	0 (0)	11 (4)

^a^This module is available to everyone as an additional module regardless of their Military-Civilian Adjustment and Reintegration Measure profile.

The “time spent” refers to the time participants spent engaging with each module. This metric could reflect multiple factors, including module complexity, participant interest, or difficulty with the content. The module with the highest median time spent by the users who did complete it was Beliefs About Civilians (67, IQR 23.4-110.6 minutes). It was followed by Finding Purpose (median time of 32, IQR 9-55 minutes), Regimentation (median time of 25, IQR 11-39 minutes), Resentment and Regret (median time of 17, IQR 0.25-33.75 minutes), Social Connection (median time of 13, IQR 5.5-20.5 minutes) and Help Seeking (median time of 11, IQR 0-31.5 minutes). The median time for the additional module Effective Communication in Civilian Life was 19 (IQR 10.75-27.25 minutes) minutes.

No statistically significant differences were observed (all *P*>.05) in relation to age, sex, service type, or type of needs (Purpose and Connection, Help Seeking, Regimentation, Resentment and Regret, and Beliefs About Civilians) between participants who had completed at least 1 module and those who had not started or were still in progress at the time of data extraction.

### Go-Beyond User Experience Survey Data

There were 94 self-report surveys assessing the Go-Beyond internet-based program completed. The survey responses provided insights regarding the program’s subjective usefulness, user engagement, the platform’s usability, and user recommendations.

### Perceived Usefulness and Satisfaction

Out of the 94 internet-based self-report surveys evaluating Go-Beyond, 80% (74/94) of respondents acknowledged the usefulness of the module. Moreover, 85% (80/94) of the users were “satisfied” or “completely satisfied” with the program’s features and components, and 79% (73/94) of survey respondents stated that they were “satisfied” or “completely satisfied” with the quality of the content. Furthermore, 86% (81/94) of survey respondents indicated a likelihood of implementing changes in their lives following the module, and 94% (88/94) reported they would recommend the program to other veterans or their family or friends.

### User Assessment of Platforms Engaging Features

The users were asked how engaging overall the modules were in terms of entertainment, interest, relatability, and interactivity of the modules (see Textbox 1 for questions). A total of 78% (73/94) of the survey respondents found the modules engaging overall. In addition, 86% (81/94) of participants found the time required to complete the program to be satisfactory.

### Platform Usability

A total of 91% (86/94) of users reported a high degree of satisfaction with the Go-Beyond Platform’s usability. The audio and visual presentations resonated or somewhat resonated with 94% (88/94) of users. In relation to the use of personal stories, videos, animations, and illustrations, users reported that they were: “Very helpful and informative” and commented that they enhanced the program: “I thought the quotes, illustrations, and videos were used really well in this module and very realistic.” And helped them self-reflect: “It was good. Made me look at myself a little and accept that it is ok to be flexible.” The survey indicated strong endorsements of the platform’s aesthetics, rated as “satisfied” and completely satisfied” by 86% (81/94) of users. Furthermore, 85% (80/94) of users were “satisfied” or “completely satisfied” with gestural design functions of the platform.

### User Recommendations

When questioned about the most beneficial features of the program, the majority of responses were related to (1) relatable psychoeducational content (ie, “The information behind emotions and why we feel them” and “The personal insights it provided along with leaving me with a sense of not being alone in feeling this way”), (2) opportunities for self-reflection (ie, “Given the option to self-reflect” and “Providing places to self-reflect on the information given”), and (3) practical strategies and techniques (ie, “strategies and thought techniques to widen perspectives and assist with positive actions”). On the other hand, the least helpful features included (1) a long reference list and links to resources (ie, “The links end up with more links that take you further and further from the main content”), (2) a lack of diversity in the personal videos (ie, “I don’t recall any useful or memorable audio clips with information or personal stories, although I can appreciate that it is not simple to source authentic, vulnerable, and engaging stories”). Furthermore, some technical issues were also identified (ie, “No previous work was recorded. I had to wait to I got access to a printer to complete the module and all my previous work had not been saved. Having a complete module button that takes you back to the home page would be great,” “Return to other courses button when completing the module would be great,” and “Better audio for the doctor videos. Very echo’y”).

## Discussion

### Principal Findings

The study aimed to assess the reach, adoption, and engagement with the “Go-Beyond, Navigating Life Beyond Service Program,” a web-based program designed to aid veterans in transitioning to civilian life, incorporating evidence-informed content on psychological and cultural factors. The program aims to provide education, build psychological insight, offer practical tools, and offer users links to available support. The findings underscore the potential value of the Go-Beyond program as a web-based initiative, leveraging digital technology to provide a personalized and easily accessible resource for veterans navigating the transition to civilian life.

Most of the Go-Beyond users were male (192/273, 70.3%), within 46-55 years of age, and served in the Army (153/273, 56%). Median years since the transition for Go-Beyond users who were discharged equaled 2.74 years, with a large proportion of users (165/273, 60.5 %) reporting being within 0-5 years from discharge. It is noteworthy that the first few years after discharge are widely recognized as an extremely vulnerable period for ex-serving members. Suicide risk, in particular, is notably high during the initial year of transitioning to civilian life [24]. In addition, evidence in the literature points out that many individuals experience reintegration difficulties within 6 years after separation [25], underscoring the importance of the program during this critical phase. Furthermore, the analysis of users’ needs highlights the need for a program that can address a wide array of transitional challenges and provide essential support during the transition process [18].

The overall conversion rate from completing the M-CARM questionnaire to signing up for the Go-Beyond program was 28.5% (273/959). Interestingly, among the analyzed factors, the study identified that having needs in Beliefs About Civilians and Purpose and Connection domains could be considered relevant predictors of engagement with the program. Users identified with these needs were up to almost 2 times more likely to sign up for Go-Beyond after completing the M-CARM survey. Interestingly, aligning with these findings, the module Finding Purpose had the highest completion rate (41/206, 20%), followed by Beliefs About Civilians (48/244, 19.7%). There is a limited number of studies providing conversion rates for internet-based, self-help programs for mental health, with the definition of uptake or adoption varying widely based on program structure and objectives [26]. A study assessing the public implementation of a web-based program for veterans dealing with risky alcohol use and PTSD, named VetChange, reported a conversion rate of 4.7% (1033/22,087) from unique homepage visits resulting in full account registrations [27]. Another study examining the impact of self-help resources on gambling behavior, found a conversion rate of 14% (5652/8083) from the program registration page to create an account. On the other hand, a study evaluating the MoodGYM program, which aims to prevent and manage symptoms of depression and anxiety, demonstrated a 42.2% (82,159/194,840) registration rate, considering the number of website visitors. Although further research is necessary to thoroughly assess ongoing participation after the initial sign-up to Go-Beyond, the conversion rate found in this study appears to be mostly higher than comparable published programs and indicates a decent level of interest and engagement among veterans.

The time spent on each module of the Go-Beyond Program indicates varying degrees of user engagement, with a notable proportion (31%) completing at least 1 assigned module. These outcomes also correspond to the findings of other studies [27]. A systematic review of digital self-help interventions for depression, low mood, or anxiety demonstrated that real-world uptake and engagement present a significant variability and may differ from adherence in controlled trials [27]. Across the studies analyzed in that research, minimal usage of the intervention ranged from 21% to 88%, with users either accessing it at least once or completing one module. However, completion or sustained use, defined as finishing all modules or the final assessment, or continuing to use the apps after a prolonged period of time was lower and ranged from 0.5% to 28.6%. Research has demonstrated that factors such as motivation, previous experience, and the perceived relevance of course content can significantly influence engagement and completion rates in online interventions [28]. Jardine et al [29] identified uncertainty about the intervention’s usefulness and usability as the main barriers to its uptake. Previous studies also revealed that forgetting about the intervention, not finding time for it, and not finding it useful posed main barriers to early engagement [28]. Further barriers to engagement included technological challenges, severe mental health issues, lack of personalization [30], lack of privacy, or difficulty in relating to the content [30,31]. Conversely, facilitators include among others user-friendly interfaces, personalized content, social connectedness facilitated by the intervention, and the perception of gaining better insight into the condition [30,31].

Beatty and Binnion [31] examined 36 studies to determine predictors for adherence to web-based interventions, such as demographic, psychological, characteristics of the presenting problem, and intervention and computer-related factors. The findings indicated that the female sex, higher treatment expectancy, sufficient time, and personalized intervention content were associated with increased adherence. Further, not having enough time, dissatisfaction with program content, perceiving content as impersonal, and computer difficulties were found to decrease adherence [32]. Sex was not found to play a significant role in predicting the completion of any module in the Go-Beyond program in our study. However, as identified in the users’ feedback, technical challenges experienced by Go-Beyond users, such as difficulties with saving completed worksheets, accessing the homepage with all relevant modules, and audio quality, may have influenced veterans’ frustration levels and their willingness or ability to complete the modules. On the other hand, the highly personalized content of the program, which offers users modules tailored to their identified M-CARM areas of need, may have contributed to the completion rates and engagement with the module evidenced by high ratings of quality of content and usefulness of the program. Moreover, the results of the user survey underscore overall user satisfaction with the Go-Beyond program, encompassing various aspects of module effectiveness, user engagement, platform usability, and user recommendations. They suggest that the Go-Beyond internet-based program engages users, providing valuable content with aesthetically pleasing and functionally efficient modules. Users also express a high likelihood of recommending the program to others, indicating a potential helpful impact on a broader audience. However, further studies evaluating specific barriers and facilitators for engagement with Go-Beyond are necessary.

While considering factors impacting adherence, it needs to be noted that Wojtowicz et al [32] suggest that levels of guidance or support through phone or email contribute to increased adherence to internet-based interventions. It is suggested that guided support may enhance motivation to participate or foster a greater sense of accountability to adhere [33]. Peer support and professional-assisted delivery of web-based interventions also pose a potential solution to the enhancement of engagement in web-based interventions [34]. Research suggests that peer-assisted internet-based interventions have proven to be feasible in the veteran population [35,36]. Since Go-Beyond was designed as a completely self-help tool, an entirely self-help format may be challenging for the ex-Defence cohort where low motivation and avoidance are key features in common mental health disorders experienced by veterans such as depression, anxiety, and PTSD [37-39].

The findings of this preliminary evaluation provide several lessons learned from the preliminary implementation and lay the groundwork for further investigation and refinement of the Go-Beyond program. In this context, it is crucial to emphasize the importance of using real-world data when evaluating adoption and engagement in digital mental health interventions. As previously highlighted, real-world settings often differ from controlled trial conditions, as individuals’ interaction with these interventions can be influenced by factors such as accessibility, usability, and personal preferences. Therefore, comparing uptake and usage patterns across various interventions using real-world data could offer invaluable insights, enabling researchers and developers to identify trends, successes, and areas for improvement in the field of digital mental health. This, in turn, would facilitate making informed decisions to enhance user experience and outcomes [26].

### Limitations and Future Directions

There are several limitations to this study. Veterans who chose to participate in the Go-Beyond program may not represent the entire veteran population, leading to selection bias. Since the M-CARM measure as well as the Go-Beyond program are available to anyone, there is a possibility that some of the users might have been members of the general population interested in the program. Those who completed the M-CARM and signed up for the Go-Beyond might have unique characteristics or experiences that differ from nonparticipants, impacting the generalizability of the findings. Therefore, the existing literature is the only source of information to draw conclusions as to why some chose to enroll in the program and complete assigned modules. Furthermore, only 31% of users who signed up for Go-Beyond completed at least 1 assigned module and provided feedback. As a result, the favorable quantitative and qualitative feedback received regarding the program may not fully represent the overall user experience. This highlights the importance of considering the perspectives of those who did not fully engage with or did not complete the program in future evaluations another limitation of our study is related to the interpretation of the “time spent” metric. While “time spent” refers to the duration participants engaged with each module, this measure does not account for various underlying factors that could influence the observed time. For instance, a longer duration may indicate that a module was more complex, more engaging, or potentially more challenging for participants. This variability can complicate the interpretation of whether differences in “time spent” are due to the inherent characteristics of the modules or other participant-specific factors.

Finally, this was a preliminary examination of the reach, user engagement, and completion rates of the Go-Beyond program, and further evaluations of the barriers and facilitators, as well as the effectiveness of the internet-based program on veterans’ readjustment to civilian life are needed.

### Conclusion

In conclusion, the Go-Beyond program may be a promising initiative in leveraging digital technology to support veterans during their transition to civilian life. The study’s outcomes contribute valuable insights into user engagement, completion rates, and the program’s overall adoption. As the program continues to evolve, ongoing evaluation and refinement will be crucial to enhancing its effectiveness and addressing the diverse needs of military personnel in their reintegration journey. Our study supports the notion that web-based interventions hold substantial promise in enhancing the experience of transition into the civilian life of veterans. Future research should examine factors that predict the use and nonuse of Go-Beyond, how use changes over time, and what factors can serve as predictors of use. Furthermore, it is crucial to incorporate relevant outcome measures to gain a thorough understanding of its effectiveness and potential benefits.

## Abbreviations

GMR: Gallipoli Medical Research
M-CARM: Military-Civilian Adjustment and Reintegration Measure
PTSD: posttraumatic stress disorder
RSLQ: Returned and Services League of Australia Queensland

## Data Availability

The datasets generated during and analyzed during this study are not publicly available due to ethical and privacy considerations but are available from the corresponding author on reasonable request.
